# Supplementary figures and images for: Optimized Protocol for Primary Rat Hepatocyte Isolation and a Model for Investigating Experimental Steatosis
Source: Methods Protoc. 2025 Sep 19;8(5):111. doi: 10.3390/mps8050111 (PMC12452565; doi:10.3390/mps8050111)

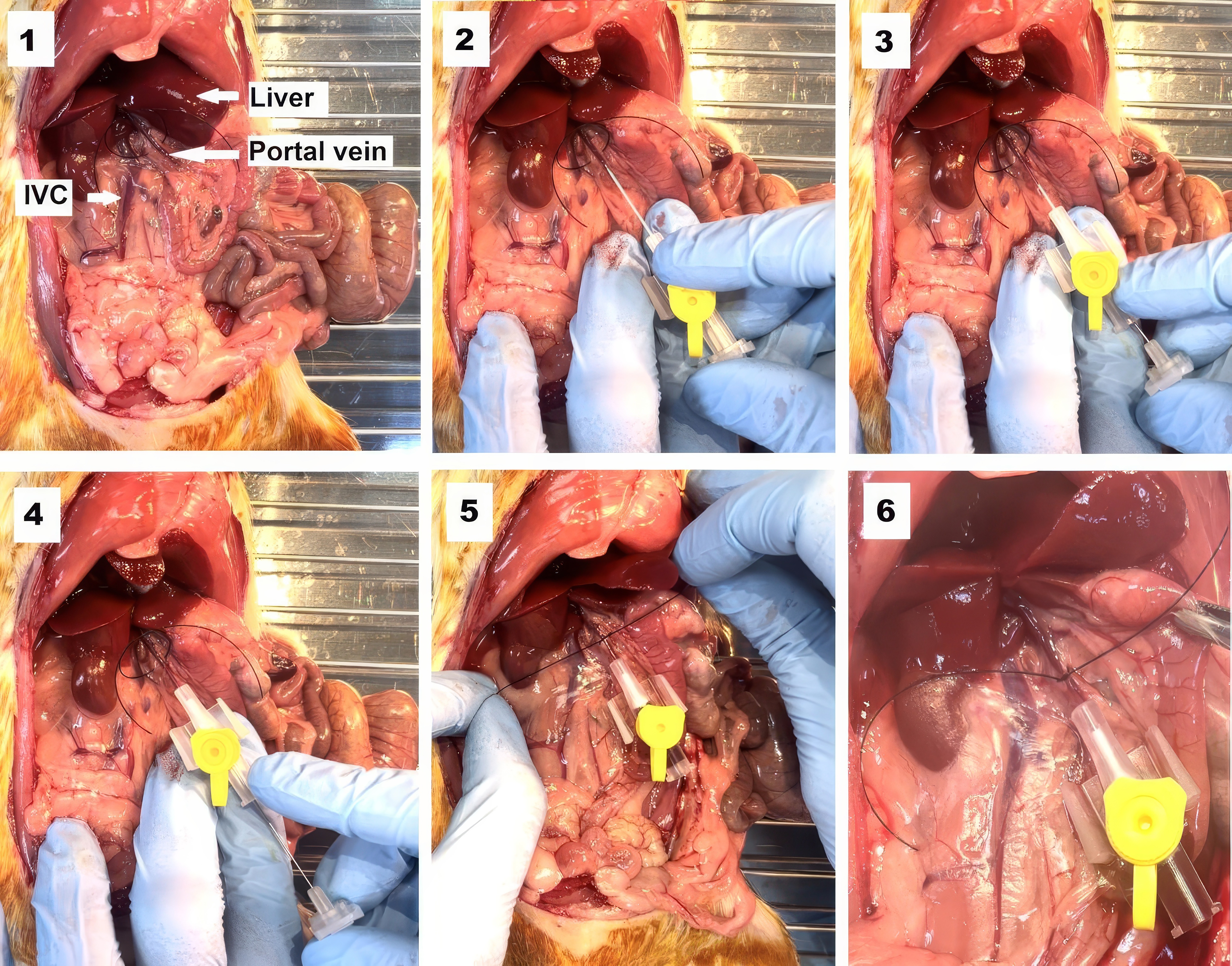

Supplement: Supplementary file 1 [file mps-08-00111-s001.zip › mps-3829525-GA.jpeg]
